# The impact of a patient decision aid on treatment choices after anterior cruciate ligament injuries

**DOI:** 10.1186/s40634-023-00633-9

**Published:** 2023-08-16

**Authors:** Hanne Mainz, Lone Frandsen, Peter Fauno, Kirsten Lomborg, Martin Lind

**Affiliations:** 1 Clinic of Sports Traumatology, Department of Orthopaedic Surgery, Aarhus University Hospital, Aarhus, Denmark; 2 Research Centre of Patient Involvement, Aarhus University Hospital, Aarhus, Denmark; 3 Department of Clinical Medicine, University of Copenhagen, Copenhagen, Denmark; 4 Department of Clinical Research, Copenhagen University Hospital – Steno Diabetes Center Copenhagen, Copenhagen, Denmark; 5 Department of Clinical Medicine, Aarhus University, Aarhus, Denmark

**Keywords:** Patient decision aid, Anterior cruciate ligament injury, Surgical treatment, Non-surgical treatment, Shared decision making, Patient-centred care

## Abstract

**Purpose:**

The present study aimed to investigate whether exposure to a patient decision aid (PDA) had an impact on the proportion of patients selecting non-surgical or surgical treatments after anterior cruciate ligament (ACL) injuries and whether exposure to a PDA affected the proportion of patients switching from non-surgical to surgical treatment within the first year.

**Methods:**

In a consecutive case series, proportions of surgery and non-surgery were compared before and after patients’ exposure to a PDA. Data were collected from the health records of patients with ACL injuries who presented to the Clinic of Sports Traumatology. To identify proportional differences between the two groups, t-tests and proportion tests were used.

**Results:**

In total, 1,053 patients with ACL injuries were included: 563 patients with no exposure to the PDA (January 2015 to January 2017) and 490 patients with exposure to the PDA (January 2017 to January 2019). Before implementing the PDA, 27% of the patients selected non-surgical treatment. After implementing the PDA, 30% choose non-surgical treatment (*p* > 0.05). Before implementing the PDA, 21% of patients who initially chose non-surgical treatment had surgery within the first year. After implementation of the PDA, this number fell to 16%, but the difference was not statistically significant (*p* > 0,05).

**Conclusion:**

Exposure to the PDA did not significantly alter the proportion of ACL injury patients selecting non-surgical or surgical treatments or the proportion of patients switching to surgery within the first year.

## Background

Anterior cruciate ligament (ACL) injuries primarily affect young athletes, leading to loss of knee function due to joint instability, which results in decreased activity and poor knee-related quality of life [10, 17, 18]. Since the introduction of surgical ACL reconstruction, ACL injuries have primarily been managed surgically using autologous tendon grafts [14]. Non-surgical treatment strategies based on systematic rehabilitation have been advocated as a potential alternative to surgery [11, 13]. Frobell et al. demonstrated in a randomized clinical trial that structured rehabilitation as first-line treatment for ACL injuries resulted in subjectively satisfactory outcomes in 50% of the patients [6]. Acceptable outcomes achieved by rehabilitation-based treatment have resulted in a widespread acceptance of non-surgical treatment for ACL injuries. The existence of two treatment concepts (surgical and non-surgical) with good outcomes has led to the need for patient decision tool when healthcare professionals, together with their patients, decide the best treatment option for ACL injuries [8].

To achieve an optimal treatment outcome, patients should have the option of involvement in the treatment decision process. One method of enabling patient involvement is to use a patient decision aid (PDA) during treatment counselling. Using a PDA in clinical situations can benefit when there is more than one relevant treatment option. All treatment options have advantages and disadvantages that patients might view differently. To ensure the best possible treatment outcome, with the treatment tailored to suit the patient’s specific needs, both the healthcare professional's and the patient's views must be considered in the treatment decision. Shared decision-making (SDM) takes account of the medical expert's competencies and the individual values of the patient [5]. The concept of SDM is an essential element of patient-centered care and has been defined as: ‘an approach where clinicians and patients share the best available evidence when faced with the task of making decisions, and where patients are supported to consider options, to achieve informed preferences’ [21]. The concept of SDM and the use of a PDA can help patients to make informed, value-based decisions, together with healthcare professionals, by assisting the patient in identifying and communicating personal values essential for the treatment options [4, 9, 21].

Recent review studies demonstrated that PDAs and SDM improve communication between patients and healthcare professionals. Patients exposed to PDAs and SDM are more knowledgeable about their treatment and have more realistic treatment expectations [1, 15]. A meta-analysis from 2017 indicated that PDAs seemed to reduce the number of elective surgeries in favour of more conservative options [21]. Within the area of musculoskeletal disorders, PDAs have been used in patients with hip and knee osteoarthritis [3]. In a systematic review, a PDA improved patient knowledge but had a limited impact on treatment choice [2]. Thus far, the impact of PDA usage on the treatment decisions of patients with ACL injuries has not been investigated.

The present study aimed to investigate whether exposure to a PDA had an impact on the proportion of patients selecting non-surgical or surgical treatments after ACL injuries and whether exposure to a PDA affected the proportion of patients switching from non-surgical to surgical treatment within the first year.

## Methods

The study was designed as a consecutive case series during a four-year period and compared the proportion of patients with ACL injuries having surgical or non-surgical treatment before and after implementing a PDA.

### Data collection

The study was conducted at a University Hospital in Denmark. The study included patients aged 15 years or older with an ACL injury who presented to the Clinic of Sports Traumatology. Patients with a previous ACL injury of the same knee, multiligament injury, or displaced meniscus injury requiring early surgery, were excluded. The included patients were divided into two groups: patients without exposure to the PDA (present in the clinic from January 2015 to January 2017) and patients with exposure to the PDA in treatment decision-making (present in the clinic from January 2017 to January 2019). Demographic data on age, sex, and treatment categorized as surgical or non-surgical were retrospectively collected from the patient’s health records. In addition, data were collected on patients who initially selected non-surgical treatment but switched to surgery within the first year from the time of injury.

### PDA

The development of the PDA was based on 12 criteria developed according to the International Patient Decision Aid Standards, described by the Ottawa Hospital Research Institute [5]. First, all healthcare professionals from the clinic were asked to list all possible issues that influence treatment decision-making regarding ACL injuries. Subsequently, 35 randomly selected patients with an ACL injury recruited from the clinic were asked to categorize and prioritize these issues. This resulted in eight top issues influencing treatment decisions: knee stability, possible effects on daily living activities, sports ability, workability, clinical results, risks, rehabilitation, and sick leave [12]. These were included in the PDA. Based on a literature review, the advantages and disadvantages of surgery/non-surgery were outlined for each issue. Best practice was described when no scientific evidence was available [12]. PDA was pilot tested by seven randomly selected patients, and minor corrections were made based on patient feedback (described in a previous article) [12].

The process of SDM and the associated PDA was then introduced to all doctors in the clinic. A project team member attended at least two consultations with each doctor to assess whether the patient and the doctor were actively involved in decision-making. Further, the member of the project team evaluated whether they shared their knowledge and treatment preferences [12]. After the consultation, the doctor and team members assessed the use of SDM and the PDA. Following these evaluations, the use of the PDA was implemented as a standard procedure for ACL patients in the clinic in January 2017.

### Statistical analyses

Descriptive statistics were used to present the patient population. To identify differences before and after exposure to the PDA, a t-test (analyzing age differences) and proportion tests were used (analyzing differences in sex and treatment option). No regression analysis was performed as the patient groups before and after exposure to the PDA were comparable. Skewness and kurtosis tests were used to test age for normality. Results were presented with proportions (%), means, CI and range. A p-value less than 0.05 was considered statistically significant. Data analysis was conducted in Stata version 17.

### Ethics

The Danish Patient Safety Authority approved the use of the data from the medical health records for the patients in this study. The need for patient consent was waived on the basis that the study had a recognizable purpose (No.: 3013–2983/1). As required by the Danish Patient Safety Authority, a healthcare professional appointed by the management of the Orthopaedic department at the hospital anonymized the patient data and transferred the data to the research team.

## Results

In total, 1,053 patients aged 15 years or older with a primary ACL injury were treated in the Clinic of Sports Traumatology during a four-year period from January 2015 to January 2019. Most patients selected surgery as the treatment option, with more male (80%) than female patients (62%) choosing this option. Patients who chose surgery were younger (mean age 24 years) than those who chose non-surgical treatment (mean age 37 years (Table 1).

**Table 1:** Demographic data

*N* = 1,053	**Non-surgical patients** (95% CI) *n* = 299	**Surgical patients** (95% CI) *n* = 754	**All patients** (95% CI)
**Females**	38% (33–42)	62% (58–66)	54% (51–57)
**Males**	20% (33–44)	80% (56–67)	46% (43–49)
**Age** (years)	37 (35–38) Range 17–64	24 (23–25) Range 15–58	28 (27–28) Range 15–64

From January 2015 to January 2017, 563 patients were treated for ACL injury in the clinic before the PDA was implemented. After implementing the PDA, 490 patients were treated for ACL injury from January 2017 to January 2019 (Table 2). There was no significant difference between the two groups regarding sex and age (Table 2). Before introducing the PDA, 27% of the patients decided to have non-surgical treatment. After introducing the PDA, this increased slightly to 30%, with no statistically significant difference between the groups (*p* > 0.05) (Table 2).

**Table 2:** Differences in patients’ treatment decisions before and after Patient Decision Aid (PDA) exposure according to sex and age

*N* = 1,053	**Before PDA**^a^ (*n* = 563) (95% CI)	**After PDA**^b^ (*n* = 490) (95% CI)	**Difference**
**Females**	44% (40–49)	47% (42–51)	3% (n.s)
**Males**	56% (51–60)	53% (49–58)	
**Age** (years)	27 (25–28)	27 (27–28)	0% (n.s)
**Non-surgical treatment**	27% (23–31)	30% (26–34)	3% (n.s)
**Surgical treatment**	73% (70–77)	70% (66–74)	

Non-significant *p* > 0.05 (n.s)

^a^From January 2015 to January 2017, ^b^ From January 2017 to January 2019

For all patients who decided initially to have non-surgical treatment, 18% had surgery within the first year after the ACL injury. Before implementation of the PDA, 21% of the patients had surgery within the first year after initially selecting non-surgical treatment. After implementation of the PDA, this number fell slightly to 16%, but the difference was not statistically significant (*p* > 0.05) (Table 3).

**Table 3:** Patients initially selecting non-surgical treatment and have surgery within the first year according to Patient Decision Aid (PDA) exposure

*N* = 299	**Non-surgical treatment** (95% CI)	**Surgical treatment** (95% CI)
**Before PDA**^a^ (*n* = 148)	79% (72–85)	21% (15–28)
**After PDA** ^b^ (*n* = 151)	84% (78–89)	16% (11–22)
**Difference**	5% (-4–14), (n.s.)*	

^*^No statistically significant difference after implementation of the PDA *p* = 0.26

^a^From January 2015 to January 2017, ^b^ From January 2017 to January 2019

## Discussion

The main finding of this study was that the use of PDA did not change the proportion of ACL injury patients selecting non-surgical and surgical treatments and the patients' decisions to switch from non-surgical to surgical treatment within the first year.

A systematic review revealed that exposure to a PDA compared to usual care reduced the number of patients choosing elective invasive surgery in favour of more conservative treatment options [20]. In many orthopaedic specialties, the number of patients undergoing surgery continues to rise, and there is some concern about whether these surgeries provide clinical benefits for all patients [16]. The primary purpose of a PDA is to ensure that patients are involved in the decision process, and SDM using a PDA did not reduce the number of patients choosing surgery in our study. The result of our study was more in line with two other studies from the orthopaedic field. One study, which included patients with knee and hip arthroplasty, concluded that the introduction of PDA was not associated with more significant shifts in patients’ treatment preferences [7]. In another study, patients on a waiting list considering total knee arthroplasty were randomized, and half of the patients were exposed to a PDA. The authors concluded in this study that PDA improved the patients’ decision quality [19]. However, the proportion of patients on the waiting list for surgery did not change after exposure to the PDA [19]. In our previous study developing the PDA, patients found that the PDA was a very useful tool. Healthcare professionals reported that the PDA improved SDM by supporting dialogue and clarifying patients’ values concerning issues relevant to treatment choices [12].

A Cochrane review from 2017 recommended more research on patient compliance with the chosen treatment option [21]. Our study did not find that the PDA altered the proportion of patients choosing non-surgical treatment and later changed their decision to surgical treatment.

This study had limitations. Data were collected during a four-year period with the assumption that the number of patients would be sufficient to identify important clinical differences (*n* = *1053*). However, the statistical power of the sub-group analyses of the non-surgical patients switching to surgical treatment was limited (*n* = 299). Further, when comparing the two groups, it would have been appropriate to control for broader patient characteristics that may influence treatment decisions, such as information about the patient's sport, professional level, working conditions, BMI, and the intensity of the rehabilitation. Unfortunately, this information was not consistently described when we reviewed the patients' medical records. Although sex and age were associated with the treatment decision, no differences were found for these patient characteristics in the two groups compared regarding PDA exposure. This could indicate that the comparison of the two groups was reasonable.

The implications are relevant to clinical practice. Using a PDA does not change the number of patients seeking ACL surgery, nor does it result in more patients requiring surgery within the first year. In conjunction with previous studies showing that patients and healthcare professionals consider PDA to be an appropriate decision-making tool in the treatment of patients with ACL injury, the adoption of PDA is recommended. Further studies are needed to investigate whether PDA may improve clinical outcomes for the patients, such as knee function, the possibility of returning to usual sports activities, and patient decision satisfaction in follow-up studies for patients with ACL injuries.

## Conclusions

Exposure to the PDA did not alter the proportion of ACL injury patients selecting non-surgical and surgical treatments or the proportion of patients switching to surgery within the first year. The result of the study indicates continued use of the PDA since patients and healthcare professionals previously have reported PDA as applicable to assist decision-making.

## Data Availability

The data underlying this article can be shared on reasonable request to the first author.

## Abbreviations

ACL: Anterior cruciate ligament
PDA: Patient decision aid
SDM: Shared decision making
CI: Confidence interval
